# Supplementary material for: A Novel CARMIL2 Immunodeficiency Identified in a Subset of Cavalier King Charles Spaniels with Pneumocystis and Bordetella Pneumonia
Source: J Fungi (Basel). 2024 Mar 5;10(3):198. doi: 10.3390/jof10030198 (PMC10970956; doi:10.3390/jof10030198)
Supplement: Supplementary file 1 [file jof-10-00198-s001.zip › jof-2887564-supplementary.pdf]

**Table S1.** Clinical data for 15 dogs previously diagnosed with PCP and clear of the *CARMIL2* variant

| **Breed** | **Age (years)** | **Sex** | **Country of Origin** | **Reference** |
|---|---|---|---|---|
| Whippet | 3 | MN | Austria | [27] |
| American Staffordshire | 5 | MN | Italy | [6] |
| Akita Inu | 2 | MI | Italy | [6] |
| Toy Poodle | < 1 | MI | Italy | [6] |
| Border Collie | < 1 | FI | Italy | [6] |
| Golden Retriever | 11 | MI | Italy | [6] |
| Not available | Not available | Not available | Italy | [6] |
| Boxer | < 1 | FI | Italy | [6] |
| Not available | Not available | Not available | Italy | [6] |
| Not available | Not available | Not available | Italy | [6] |
| Not available | Not available | Not available | Italy | [6] |
| English Bulldog | 11 | MI | Italy | [6] |
| Jack Russel Terrier | 10 | FI | Italy | [6] |
| Mixed breed | 11 | FI | Italy | [6] |
| Not available | Not available | Not available | Italy | [6] |

FI = Female intact; MI = Male intact; MN = Male neutered
